# Comparison of Development and Antioxidative Ability in Fertilized Crossbred (Yorkshire × Landrace × Duroc) Oocytes Using Duroc and Landrace Sperm

**DOI:** 10.3390/ani14243562

**Published:** 2024-12-10

**Authors:** Hayoung Lee, Hyewon Kim, Jisoon An, Hee-Tae Cheong, Sang-Hee Lee

**Affiliations:** 1 College of Animal Life Sciences, Kangwon National University, Chuncheon 24341, Republic of Korea; hayoung0721@kangwon.ac.kr (H.L.); embryo@kangwon.ac.kr (H.K.); ajss95@kangwon.ac.kr (J.A.); 2 College of Veterinary Sciences, Kangwon National University, Chuncheon 24341, Republic of Korea; htcheong@kangwon.ac.kr; 3 School of Information and Communications Technology, University of Tasmania, Hobart, TAS 7005, Australia

**Keywords:** Duroc, embryo development, Landrace, sperm characteristics

## Abstract

Pig production through crossbreeding methods is a pillar of the swine industry; however, research on the fertilization ability of male pigs in crossbreeds is lacking. Therefore, this study investigated the effects of Duroc sperm (DS) and Landrace sperm (LS) on fertility in Yorkshire × Landrace × Duroc (YLD) oocytes. Sperm were collected from the Duroc and Landrace species, and sperm characteristics, viability, and acrosome reactions were analyzed using flow cytometry. Oocytes were collected from YLD ovaries, and the fertility of DS and LS was determined using in vitro fertilization (IVF). Reactive oxygen species (ROS) and antioxidative abilities were analyzed using H_2_DCFDA and a Cell Tracker Red assay. Pluripotency (*OCT4*, *SOX2*, and *NANOG)*, antioxidative (*SOD1*, *SOD2*, *CAT*, and *GPx1*), apoptotic (*Bax* and *Bcl-2*), and cell cycle-related (*Cdc2* and *CCNB1*) genes were detected using quantitative reverse transcription polymerase chain reaction (qRT-PCR) in oocytes fertilized with sperm. The results showed no significant difference in viability or acrosome reaction between DS and LS. ROS levels were significantly lower in the LS group than in the DS group, whereas glutathione (GSH) levels in the embryo did not significantly differ between the DS and LS groups. The *OCT4*, *GPx1*, and *Cdc2* mRNA expression levels were significantly higher in the LS than DS groups. Blastocyst formation was significantly higher in the LS than DS groups. ROS levels were reduced, and blastocyte formation was increased in LS-obtained embryos. In conclusion, these results provide a fundamental understanding of using Landrace semen in the three-way crossbreeding of YLD pigs.

## Simple Summary

The use of male semen is critical in pig breeding. For meat production, three-way crossbred Yorkshire × Landrace × Duroc (YLD) has commonly been used; however, reproductive data on the outcomes of crossbreeding between purebred males and crossbred females in pigs remain lacking. Therefore, we compared the fertility of Duroc sperm (DS) and Landrace sperm (LS) when used for the fertilization of YLD oocytes. The cleavage of DS and LS was similar in fertilized oocytes; however, the blastocyst rate was increased by LS. Moreover, our findings confirmed that the expression of genes associated with pluripotency, antioxidant enzymes, and cell cycle was upregulated in embryos fertilized with LS. These results suggest that LS improves the fertilization rate in three-way crossbred YLD female pigs.

## 1. Introduction

The swine industry, which has advanced in various fields, including reproduction, genetics, disease prevention, and production management [1,2], provides stable supplements to animal protein and biomedical models for humans. Several porcine species are used for the breeding and making of pork, including Yorkshire, Landrace, and Large White, which are widely used for breeding piglets, have excellent fertility, and maternal behavior for reproduction [3,4]. The characteristics of each variety can be compared and analyzed, and the preferred array can be selected for production [5]. Additionally, combining the excellent qualities of both breeds through crossbreeding with crossbred sows could provide a model for developing diversity and improving production efficiency in the breeding of pigs [6].

The Duroc is widely used as the primary sire breed since it is characterized by superior muscle development, high meat quality, and rapid growth [7]. Pigs used for pork production are typically the offspring of a three-way crossbreeding system, Yorkshire × Landrace × Duroc (YLD), utilizing Landrace and Yorkshire sows inseminated with Duroc boar semen in the livestock industry [8]. For successful breeding improvements, it is essential to evaluate the ability of female and male gametes to produce purebred and crossbred terminal lines [9]. Specifically, comparing the sperm from Duroc and Landrace boars is critical as these breeds represent distinct genetic contributions and reproductive characteristics, which are pivotal for optimizing terminal crossbreeding strategies and enhancing overall breeding efficiency [10].

Considerable variability has been reported in the fertility and reproductive efficiency of crossbred sows produced from crossbred Pietrain and Duroc × Pietrain boar terminal lines [11]. Assessing the conception rate through artificial insemination (AI) is the best diagnostic method to accurately verify sperm fertility according to male species under in vivo conditions; however, this method is time-consuming, labor-intensive, and cannot be used to analyze embryo quality after fertilization. Therefore, it is necessary to use in vitro fertilization (IVF) technology to accurately analyze the quality of embryos fertilized with sperm, depending on the boar species.

IVF includes ovarian stimulation, oocyte retrieval, fertilization, embryo culture, and embryo transfer to the uterus, with fertilization achieved either by co-culturing the oocyte and sperm or through a procedure called intracytoplasmic sperm injection (ICSI) [12]. In addition, IVF research has been conducted in various fields, such as humans [13], mice [14], sheep [15], cows [16], and pigs [17]. IVF technology is used in multiple ways in the swine industry, such as producing high-quality embryos through breeding programs [18], selective breeding through genetic characteristics [19], and analyzing mechanisms through reproductive physiology research [17]. Additionally, a previous study assessed fertilization and embryo development at two different sperm–oocyte ratios using pigs from the Duroc and Landrace breeds [20]. Sperm morphological parameters in semen used for artificial insemination showed different proportions depending on the variety [21]. Various studies have attempted to increase the efficiency of embryo development and improve embryo quality during IVF; however, this depends on the semen characteristics of each pig breed. Therefore, to achieve efficient production of high-quality embryos derived from in vitro production, a comparative analysis of the semen used for IVF is necessary. IVF experiments to determine semen capacity can be used to assess fertilization capacity. Competency assessments should be made, especially for the Duroc and Landrace varieties used primarily in economic animals.

The quality of ejaculated sperm is generally evaluated based on its volume, concentration, viability, and motility for use in artificial insemination. The concentration [22], viability [23], and motility [24] of mammalian semen highly correlate with fertilization. Moreover, sperm head size, tail size, and concentration vary among species; these indicators are related to fertilization capacity. Although differences in semen quality between Duroc and Landrace boars have been reported [25], data on the fertility of Duroc and Landrace boar sperm in Landrace × Yorkshire sows bred for pork production in the swine industry remain lacking. This necessitates further verification of the specific capabilities of semen according to breed through in vitro research that can precisely evaluate its fertilization capacity.

Oxidative stress is closely related to sperm characteristics, such as plasma membrane integrity, acrosome reaction, and the antioxidative ability of fertilized oocytes, interrupting successful fertilization in pigs [26]. Among the Landrace and Duroc breeds, sperm with a lower antioxidant capacity to defend against oxidative stress are more likely to experience DNA fragmentation, negatively affecting fertilization capability and resulting in smaller litter sizes [10]. In embryos fertilized in vitro, cleavage and metabolic activities are affected by oxidative stress during development. Previous studies have confirmed that inhibiting oxidative stress during this process can improve embryo development [27,28]. Therefore, evaluating the oxidative stress and antioxidant capacity of in vitro embryos is essential for assessing their development and quality.

Several physiological differences exist between Duroc and Landrace pigs, yet there is a lack of reproductive cells in both breeds [29]. Additionally, there is a lack of information on the molecular impact of damaged sperm DNA on fertilization and subsequent embryo development [30]. Understanding the changes in the relative mRNA abundance of specific genes can provide insights into sperm DNA damage and improve embryo quality. Therefore, characterizing the sperm from Duroc and Landrace pigs enhances their usability.

Therefore, this study compared the fertility of Duroc and LS by analyzing plasma membrane integrity and the acrosome reaction in Duroc and Landrace boar sperm, as well as in vitro fertility, antioxidative capacity, apoptosis, and cell cycle gene expression in embryos fertilized with YLD oocytes and Duroc and Landrace boar sperm.

## 2. Materials and Methods

### 2.1. Sample Preparation

A total number of 20 ejaculates were collected from Duroc (n = 10) and Landrace (n = 10) using the gloved-hand method (Gumbo Artificial Insemination Industry, Wonju, Republic of Korea). The age of the experimental five Duroc and Landrace were 25.6 ± 4.2 and 28.3 ± 7.9 months, respectively. For the analysis of sperm characteristics, the collected DS and LS were diluted with modified Modena B to 1.0 × 10^7^ sperm/mL, as previously described [31]. Only ejaculates with >80% total motility, 60% plasma membrane integrity, and <20% acrosome reaction were used in the experiment, and the samples were transported to the laboratory within 2 h at 18 °C [31,32].

### 2.2. Sperm Characteristics

Fluorescent staining was performed using the LIVE/DEAD Sperm Viability Kit (Invitrogen, Carlsbad, CA, USA) to assess boar sperm membrane integrity [33]. Briefly, the final concentrations of SYBR14 and propidium iodide (PI, Sigma, St. Louis, MO, USA) were 1.0 nM and 1.2 µM, respectively. The acrosome reaction was assessed after staining sperm with peanut agglutinin conjugated with phycoerythrin (FITC-PNA, Sigma) and PI double staining [34]. The final concentrations of FITC-PNA and PI were 2.0 and 1.2 µM, respectively. To analyze intracellular hydrogen peroxide (H_2_O_2_) and glutathione (GSH) levels, samples were incubated with 0.5 μM 2′,7′-Dichlorodihydrofluorescein diacetate (H_2_DCFDA, Invitrogen) and 1.0 μM CellTracker Red CMTPX (Invitrogen) for 30 min at 38.5 °C, following which 10,000 sperm per sample (n = 10) were analyzed using flow cytometry (FACSymphony, BD BioSciences, Billerica, MA, USA).

### 2.3. In Vtiro Fertilization (IVF)

The ovaries were obtained from a local slaughterhouse and transported to the laboratory within 2 h. The ovaries were collected from pigs prior to prepuberty. Cumulus–oocyte complexes (COCs) were aspirated from 3 to 6 mm antral follicles using an 18-gauge syringe and collected in phosphate-buffered saline with polyvinyl alcohol (PBS-PVA) under a stereomicroscope (SZX16, Olympus, Tokyo, Japan). The COCs were then pre-incubated in tissue culture medium-199 (TCM-199), supplemented with 0.5 µg/mL follicle-stimulating hormone (FSH, Sigma), 10 ng/mL epithelial growth factor (EGF, Sigma), 10 IU/mL human chorionic gonadotropin (hCG, Intervet, the Netherlands), and 10% porcine follicular fluid (pFF) at 38.5 °C and 5.0% CO_2_ for 22 h [35]. The COCs were matured in TCM-199 medium for 22 h and then cultured in the same medium without FSH and hCG at 38.5 °C with 5.0% CO_2_ for 22 h. Collected DS (n = 10) and LS (n = 10) were used for IVF, and samples with >80% motility and 60% viability were used; motility and viability were assessed as previously described [31].

The samples were centrifuged at 1500 rpm for 5 min at room temperature and washed with modified Modena B [36]. After removing the supernatant, the final sperm concentration was adjusted to 5.0 × 10^6^ sperm/mL and added to a modified Tris Buffer Medium (mTBM) containing caffeine. Mature COCs were segregated from the cumulus complex using 0.1% hyaluronidase (Sigma) and transferred to mTBM without caffeine. A 50 µL sperm suspension was added to 50 µL of culture medium containing the oocytes and co-cultured at 38.5 °C and 5% CO_2_ for 5–6 h. Fertilized YLD oocytes (YLDO) with DS (DS+YLDO, n = 537, 10 replications) and LS (LS+YLDO, n = 574, 10 replications) were washed with porcine zygote medium-3 (PZM-3); any remaining residues, such as sperm and cumulus cells, were removed using a glass pipette. The fertilized oocytes were transferred to 100 µL of PZM-3 and cultured at 38.5 °C and 5% CO_2_ for 48 h, following which the PZM-3 was changed, and fertilized oocytes (embryos) were cultured for an additional 120 h. The average cleavage and blastocyst formation of the embryos were analyzed under a stereomicroscope 192 h post-fertilization.

### 2.4. Detection of Intracellular H_2_O_2_ and GSH in Embryos

Intracellular H_2_O_2_ was detected via H_2_DCFDA staining in DS+YLDO (n = 66) and LS+YLDO (n = 63) groups. The embryos were incubated in 0.5 μM H_2_DCFDA for 30 min at 38.5 °C and 5% CO_2_. To confirm the GSH, the DS+YLDO (n = 35) and LS+YLDO (n = 47) were stained in 1.0 μM CellTracker Red CMTPX (Invitrogen) for 30 min at 38.5 °C and 5.0%. After staining, the embryos were washed three times with PBS-PVA and imaged using an EVOS M7000 imaging system (Invitrogen). For fluorescence staining, embryo images were captured using green (482/524 nm for intracellular H_2_O_2_) and red (542/593 nm for GSH) fluorescence filters. Fluorescence intensity was analyzed using ImageJ software version 1.54 (NCBI, Bethesda, MD, USA).

### 2.5. Gene Analysis

DS+YLDO and LS+YLDO were collected at 192 h post-fertilization. The collected embryos (50 per sample and a total of six replications) were immediately transferred to liquid nitrogen and stored at −80 °C for later experiments. Total RNA was extracted using the RNAqueous™-Micro Total RNA Isolation kit (Thermo Fisher Scientific, Waltham, MA, USA). The mRNA concentration was measured using EzDrop 1000 (Blue-Ray Biotech, New Taipei City, Taiwan). cDNA was synthesized using the PrimeScript™ 1st strand cDNA Synthesis Kit (TaKaRa, Tokyo, Japan), and reverse transcription was performed at 45 °C for 60 min after 95 °C for 5 min according to the manufacturer’s instructions [37]. Antioxidant enzyme genes (*SOD1*, *SOD2*, *CAT*, and *GPx2*), pluripotency genes (*Oct4*, *Nanog*, and *Sox2*), cell cycle-related genes (*Cdc2* and *CCNB1*), and apoptosis-related genes (*Bax* and *Bcl-2*) were analyzed using qRT-PCR (Table 1). The 1.0 µL synthesized cDNA was used to conduct PCR according to the conditions (Table 1) using a PCR premix kit (Bioneer, Seoul, Republic of Korea). Amplified products were separated using 1.5% agarose gel with RedSafe Nucleic Acid Staining Solution (INtRON Biotechnology, Seoul, Republic of Korea) electrophoresis at 100 V for 20 min and photographed under UV illumination. Relative expression of mRNA was normalized to *β-actin*, and ImageJ (NCBI) was used for image analysis.

### 2.6. Blastocyst Cell Number

The produced blastocysts by DS (DS-B, n = 27) and LS (LS-B, n = 36) at 192 h post-fertilization were fixed for 15 min in 4.0% paraformaldehyde at room temperature, stained with 1.0 µL/mL Hoechst 33342 (Sigma) for 30 min at 38.5 °C and 5.0% CO_2_, and washed three times with PBS-PVA. The intensity was measured using a fluorescence microscope (EVOS M7000, Invitrogen) at 357/447 nm. Fluorescent image data analysis was performed using ImageJ software (NCBI).

### 2.7. Statistical Analysis

Data are presented as mean ± standard error of the mean (SEM). The collected data were analyzed using Student’s *t*-test with statistical analysis system software (SAS version 9.3, SAS Institute, Cary, NC, USA). Differences were considered significant when the probability of occurrence by chance was <5% (*p* < 0.05).

## 3. Results

### 3.1. Comparison of Sperm Characteristics Between Duroc and Landrace Sperm

**Figure 1:**
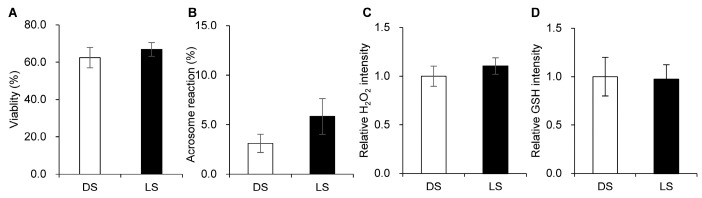
Comparison of viability (**A**), acrosome reaction (**B**), intracellular hydrogen peroxide (H_2_O_2_) (**C**), and glutathione (GSH) (**D**) levels between Duroc sperm (DS) and Landrace sperm (LS). Data are presented as the mean ± standard error mean (n = 10).

The sperm characteristics (viability, acrosome reaction, intracellular H_2_O_2_, and GSH) of the Duroc and Landrace breeds are shown in Figure 1. No significant differences in viability (Figure 1A), acrosome reaction (Figure 1B), intracellular H_2_O_2_ (Figure 1C), or GSH (Figure 1D) between DS and LS were observed.

### 3.2. Comparison of Fertility and Blastocyst Formation in Fertilized Crossbred Oocytes with Duroc and Landrace Sperm

Fertility and blastocyst formation rates of the DS+YLDO and LS+YLDO groups are shown in Table 2. We observed no significant difference in the cleavage between the DS+YLDO and LS+YLDO groups; however, the four cell stage of LS+YLDO was significantly higher than that of LS+YLDO (*p* < 0.05), and blastocyst formation was significantly higher in LS+YLDO than in DS+YLDO (*p* < 0.05).

### 3.3. Evaluation of Antioxidative Enzyme Genes, Intracellular H_2_O_2_, and GSH in Fertilized Crossbred Oocytes by Duroc and Landrace Sperm

**Figure 2:**
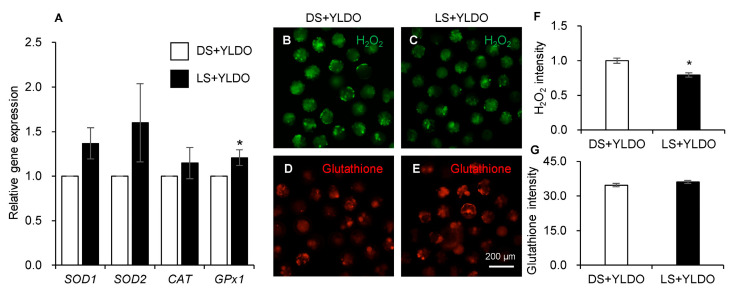
Antioxidative ability of fertilized Yorkshire × Landrace × Duroc (YLD) oocytes with Duroc sperm (DS+YLDO) and Landrace sperm (LS+YLDO). Antioxidative enzyme genes (*SOD1*, *SOD2*, *CAT*, and *GPx1*) were detected in DS+YLDO (n = 7) and LS+YLDO (n = 7) after in vitro fertilization (**A**). Intracellular hydrogen peroxide (H_2_O_2_) and glutathione (GSH) intensities in in vitro produced embryos. The intracellular H_2_O_2_ level of DS+YLDO ((**B**), n = 66) and LS+YLDO ((**C**), n = 63) was detected by H_2_DCFDA staining and glutathione of DS+YLDO ((**D**), n = 35) and LS+YLDO ((**E**), n = 47) was confirmed by CellTracker Red. Intensity of H_2_O_2_ (**F**) and GSH (**G**) was normalized and compared between DS+YLDO and LS+YLDO in pigs. Data are presented as the mean ± standard error mean. * Values with different superscript letters indicate significant differences between DS+YLDO and LS+YLDO groups (*p* < 0.05).

The antioxidative enzyme genes, intracellular H_2_O_2_, and GSH in the DS+YLDO and DS+YLDO groups are shown in Figure 2. *GPx1* mRNA was significantly higher in the LS+YLDO than DS+YLDO groups (*p* < 0.05), whereas *SOD1*, *SOD2*, and *CAT* mRNAs showed no significant expression (Figure 2A). The intracellular H_2_O_2_ intensity (Figure 2B,C, green) was significantly (*p* < 0.05) lower in the LS+YLDO than DS+YLDO groups (Figure 2F); however, there was no significant difference in GSH levels (Figure 2D,F, red; Figure 2G).

### 3.4. Differential Gene Expression in Fertilized Crossbred Oocytes with Duroc and Landrace Sperm

**Figure 3:**
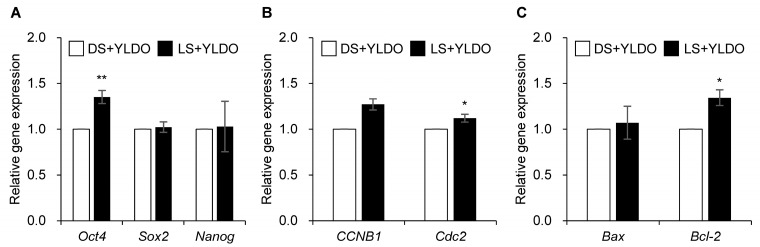
Relative expression of pluripotency genes (*Oct4*, *Sox2*, and *Nanog*) (**A**), cell cycle-related genes (*CCNB1* and *Cdc2*) (**B**), and apoptotic genes (*Bax* and *Bcl-2*) (**C**) in fertilized YLD oocytes with Duroc sperm (DS+YLDO) and Landrace sperm (LS+YLDO) in pigs. Data are presented as the mean ± standard error mean (n = 7). Values with different superscript letters indicate significant differences between DS-E (white bar) and LS-E (black bar) groups (* *p* < 0.05, ** *p* < 0.01).

The expression of *Oct4* mRNA was significantly higher in the LS+YLDO than DS+YLDO groups (*p* < 0.05); however, for the other pluripotency genes, *Sox2* and *Nanog*, mRNAs did not significantly differ (Figure 3A). In addition, the expression of *Cdc2* mRNA was significantly increased in the LS+YLDO group (*p* < 0.05); however, *CCNB1* mRNA levels did not differ between the DS+YLDO and LS+YLDO groups (Figure 3B). Lastly, the expression of *Bcl-2* mRNA was significantly improved in the LS+YLDO group compared with that in the DS+YLDO group (*p* < 0.05). In contrast, the expression of *Bax* mRNA did not change (Figure 3C).

### 3.5. Evaluation of Blastocyst Cell Numbers in In Vitro Produced Embryos

**Figure 4:**
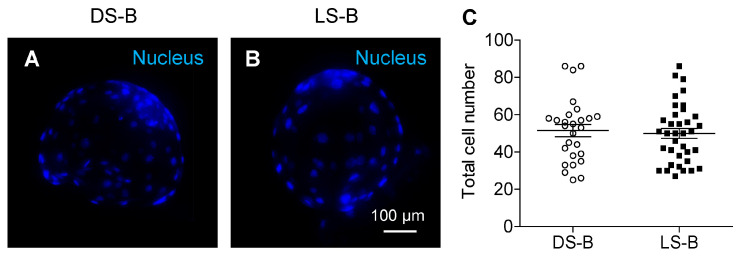
Blastocyst cell numbers in in vitro produced embryos with Duroc sperm (DS-B) and Landrace sperm (LS-B) in pigs. Hoechst 33342 was used to stain the nucleus in DS-B (**A**) and LS-B (**B**). Comparison of blastocyst cell numbers between DS-B (n = 27) and LS-B (n = 36) in pigs (**C**). Data are presented as the mean ± standard error mean.

A comparison of the total number of blastocysts produced in vitro is shown in Figure 4. The total cell number of blastocysts showed no significant difference (Figure 4C) between DS-B (51.56 ± 3.26 cells, Figure 4A) and LS-B (49.97 ± 2.65 cells, Figure 4B) in pigs.

## 4. Discussion

The analysis of sperm characteristics is critical for determining the success of implantation and pregnancy following IVF [9,11]. It plays a key role in embryonic development and overall reproductive potential, with sperm characteristics selectively managed by breeders to meet breeding and meat production goals. Therefore, in this study, we compared sperm characteristics and fertility between the Duroc and Landrace, as these two species are representative sites for breeding in the swine industry. We verified the fertility of DS and LS from the perspective of embryo development based on antioxidant activity, pluripotency, apoptosis, and cell cycle properties using IVF techniques.

Individual boars have different fertilization, cleavage, and blastocyst formation rates when fresh and freeze-thawed sperm are fertilized with oocytes [20]. In the present study, we collected sperm from Duroc and Landrace pigs and bred them to supply semen for artificial insemination to nearby pig farms. We confirmed no difference in viability, acrosome reaction, intracellular H_2_O_2_, or GSH in semen derived from 20 boars (Duroc = 10 and Landrace = 10) when >10,000 sperm per sample were analyzed using flow cytometry. A previous study reported differences in semen volume and concentration between Duroc and Landrace but not in sperm motility [25]. Similarly, we found no significant differences in sperm characteristics between the Duroc and Landrace groups. However, it is important to note that sperm viability, acrosome reaction, intracellular H_2_O_2_, and GSH levels alone do not fully represent fertility potential. While flow cytometry is effective for high-throughput quantitative analysis, it may not capture the functional aspects of sperm or their interactions with oocytes, which are essential for fertilization and subsequent embryonic development. To address these limitations, future studies should integrate complementary techniques such as sperm chromatin structure assays (SCSA) to evaluate DNA integrity or advanced imaging methods such as live-cell imaging and time-lapse microscopy to investigate sperm–oocyte interactions in greater detail. Additionally, we utilized the IVF system to assess indicators of embryo developmental ability, providing deeper insights into fertility-related parameters.

Traditionally, purebred females, such as Landrace, Large White, and Yorkshire, and purebred males, such as Duroc, Landrace, and Pietrain, have been widely used for breeding pork [8]. The genetic diversity of each breed has influenced variations in breeding, reproduction, nutrition, and meat quality [38,39]. Recently, crossbreeds have been used to produce growing–finishing pigs in the swine industry; however, studies investigating the reproductive physiology of crossbred females and purebred males utilizing their reproductive cells are limited. Therefore, comparative studies on fertility using purebred semen remain warranted to increase production efficiency in the swine industry, as YLD is one of the most commonly used species in producing growing–finishing pigs. While improved reproductive performance achieved through artificial insemination, such as litter size and litter weight at birth, has been reported [11,40], data on the developmental capacity, antioxidant potential, and genes associated with embryo development in fertilized oocytes with sperm derived from crossbred females (YLD) and purebred males (Duroc and Landrace) are lacking. In this study, although there were no differences in sperm characteristics according to breed, embryo development and blastocyst formation in YLD oocytes differed according to DS and LS.

Moreover, fertilized oocytes showed differing antioxidant capacities and developmental gene expression according to sperm type. This indicates that, apart from the characteristics of purebred sperm, other factors influence embryonic development when fertilized with oocytes derived from crossbreeds. While this study primarily focused on sperm-derived factors, variables such as media composition, incubation conditions, and external stressors cannot be excluded as potential contributors to embryonic development outcomes. Although we did not confirm differences in the genotypes of DS and LS from the perspective of genetics, we intend to investigate embryo production efficiency according to the genetic traits of purebred semen using techniques such as whole sequencing.

Embryos and blastocysts created via IVF are intricately linked to fertility. The normal cleavage process of pre-implantation embryos encompasses a complex series of events directly influencing their ability to develop into blastocysts [41,42]. In this study, we performed IVF using DS and LS sperm with oocytes from YLD to assess embryo development and blastocyst formation. There was no difference in the cleavage rate and total cell number of blastocysts between DS and LS; however, blastocyst formation was increased in DS. Generally, many factors are involved in embryo development and blastocyst formation; for example, pluripotency, antioxidative capacity, apoptosis factors, and cell cycle level are directly related to embryo development [41,42]. Therefore, we investigated the reason for the increased blastocyst formation rate of YLD oocytes by LS by analyzing the antioxidative capacity and genes associated with pluripotency, antioxidative enzymes, apoptosis, and the cell cycle in fertilized embryos. Surprisingly, we obtained positive results for antioxidative enzyme gene expression and intracellular H_2_O_2_ levels in the LS+YLDO group. As byproducts of embryo development, superoxide (O_2_^−^) and hydrogen peroxide (H_2_O_2_) affect reproductive potential [43]. This indicates that the balance between reductive and oxidative homeostasis plays a key role in regulating embryonic development [44], and various antioxidative enzymes play an important role in maintaining the intracellular redox state to protect cells against harmful effects caused by oxidative stress [45]. Herein, the overall antioxidative enzyme (*SOD1*, *SOD2*, *CAT*, and *GPx1*) genes showed a tendency to increase. Of these, *GPx1* was significantly increased in fertilized YLD oocytes when LS was used. Generally, glutathione peroxidase 1 (*GPx1*) is abundant in the cytoplasm of nearly all mammalian cells, whose preferred substrate is H_2_O_2_, a reactive oxygen species (ROS), and the balance between reductive and oxidative homeostasis is directly related to blastocyst formation in mammals [46]. Interestingly, intracellular H_2_O_2_ in fertilized YLD oocytes was significantly decreased by LS. These findings suggest that using LS enhances the developmental potential of embryos and improves their resilience to oxidative stress in three crossbred species (YLD). In the GSH detection assay using CellTracker Red CMTPX, no significant differences were observed between the two treatment groups (YLDO and LS+YLDO). We found no correlation between the antioxidant capacity of the sperm and that of the in vitro-produced embryos. In future studies, we intend to determine the influence of DS and LS on embryo development using various methods that can directly detect intracellular glutathione peroxidase.

The embryo is a cell with pluripotency, and through this characteristic cell cycle-related signaling pathways are activated along with anti-apoptosis mechanisms; these complex, interrelated processes lead to successful embryo development and blastocyst formation [47]. *Oct4* is crucial during early embryonic development, working in conjunction with *Sox2* and *Nanog* to activate pluripotency factors while playing broader regulatory roles in embryogenesis [48]. In this study, a pluripotency gene, *Oct4*, was upregulated in YLD oocytes fertilized with LS. Furthermore, we observed upregulation of the anti-apoptotic gene (*Bcl-2*) and the cell cycle-regulating gene (*Cdc2*) in the LS+YLDO group. From a cell biology perspective, *Cdc2* phosphorylates anti-apoptotic factors, such as *Bcl-2* and *Bcl-xL*, during mitosis, which, when sustained, can lead to their inactivation and induce apoptosis [43,44]. Our gene expression data support the conclusion that LS helps to reduce G2/M phase cell cycle arrest and mitigate apoptosis in porcine embryos. These results suggest that they contribute to blastocyst formation in oocytes fertilized with LS. In addition, *CCNB1* and *Cdc2* play crucial roles as key regulators of the cell cycle in mammals. Cleavage based on cell cycle upregulation actively occurs in embryos, an essential physiological phenomenon for successful blastocyst formation [49]. In our study, the expression of *Cdc2* was significantly higher in the LS+YLDO group. These results highlight the importance of *Cdc2* as a potential marker of enhanced developmental competence in the LS group. This indicates that the genes differentially expressed during the development and blastocyst formation of oocytes fertilized using DS and LS play an important role in embryonic development, and the sperm derived from Landrace helps to improve blastocyst formation in crossbred (YLS) oocytes.

The primary limitation of this study is its focus on sperm-derived factors, without fully considering the potential influence of oocyte quality, such as cytoplasmic maturity and mitochondrial function, which are critical for embryonic development. Additionally, environmental variables, including culture conditions and external stressors, were not systematically evaluated, which may have impacted the observed outcomes. Future studies should integrate comprehensive analyses of these factors to provide a more holistic understanding of the mechanisms influencing fertility and embryonic development.

## 5. Conclusions

In conclusion, our study confirmed that LS improved blastocyst formation and the expression of various genes (*GPx1*, *Oct4*, *Cdc2*, and *Bcl-2*) in YLD oocytes when used for fertilization. This suggests that the differences between DS and LS are influenced by physiological characteristics, which influence fertility outcomes. Future research could provide deeper insights into breed-specific fertility traits by extensively exploring sperm-derived factors and their potential roles in influencing embryonic development. These findings provide valuable insights into reproductive technologies and highlight the need for optimal sperm selection to obtain high-quality embryos in the swine industry. Furthermore, this study provides fundamental knowledge about fertility and the differential impact of oxidative stress, gene expression, and embryonic developmental stages across breeds, as well as the development of breed-specific fertility in the crossbreeding field of the swine industry.

## Figures and Tables

**Table 1:** Information on primer conditions.

Genes	Sequence (5′–3′)	Annealing Temp. (°C)	Cycle	Product Size (bp)	Accession No.
*Oct4*	F: AAGCAGTGACTATTCGCAAC	60	40	136	NM_001113060.1
	R: CAGGGTGGTGAAGTGAGG				
*Nanog*	F: TTCCTTCCTCCATGGATCTG	60	40	214	NM_001129971.1
	R: ATCTGCTGGAGGCTGAGGTA				
*Sox2*	F: CGCAGACCTACATGAACG	60	40	103	NM_001123197.1
	R: TCGGACTTGACCACTGAG				
*SOD1*	F: TGCAGGTCCTCACTTCAATC	60	40	257	NM_001190422.1
	R: CCAAACGACTTCCAGCATTTC				
*SOD2*	F: GTTGGCTCGGTTTCAACAAG	60	40	212	NM_214127.2
	R: GGCGTATCGCTCAGTTACAT				
*CAT*	F: CGAAGGCGAAGGTGTTTG	52	40	374	NM_214301.2
	R: AGTGTGCGATCCATATCC				
*GPx1*	F: GGACTACACCCAGATGAATGAG	60	40	216	NM_214201.1
	R: AAGAGCGGGTGAGCATTT				
*Bax*	F: CTCAGGATGCATCTACCAAGAA	60	40	213	XM_003127290.5
	R: GCACCAGTTTACTGGCAAAG				
*Bcl-2*	F: AACTTGGATGGCCACTTACC	60	40	199	NM_214285.1
	R: TTTCCGACTGAAGAGCGAAC				
*Cdc2*	F: GGTGTTCCTAGTACTGCCATTC	60	40	179	NM_001159304.2
	R: GAATCCATGAACTGACCAGGAG				
*CCNB1*	F: GTGTCAGGCTTTCTCTGATGT	62	40	199	NM_001170768.1
	R: CCAGTCAATTAGGATGGCTCTC				
*β-actin*	F: GGACTTCGAGCAGGAGATGG	60	40	233	XM_003124280
	R: GCACCGTGTTGGCGTAGAGG				

**Table 2:** Comparison of in vitro fertility and blastocyst formation in oocytes of Yorkshire × Landrace × Duroc (YLD) with Duroc and Landrace sperm in pigs.

Group	No. of Oocytes	Cleavage (%)	No. of Embryo Development to (%)				
			2 Cell	4 Cell	8 Cell	>16 Cell	Blastocyst
DS+YLDO ^1^	537	460 (84.83 ± 3.64)	29 (5.78 ± 1.14)	19 (3.38 ± 0.47)	86 (16.02 ± 3.38)	258 (48.28 ± 5.83)	68 (15.18 ± 3.64)
LS+YLDO ^2^	554	470 (84.4 ± 3.09)	25 (4.54 ± 0.97)	34 (6.44 ± 1.16) *	80 (13.38 ± 3.53)	225 (42.63 ± 5.15)	106 (17.41 ± 3.54) *

Data are presented as the mean ± standard error mean (n = 10). * Values with different superscript letters indicate significant differences within the same row (*p* < 0.05). ^1^ Fertilized YLD oocytes with Duroc sperm (DS+YLDO), ^2^ fertilized YLD oocytes with Landrace sperm (LS+YLDO).

## Data Availability

All data generated or analyzed during this study are included in this published article.
